# Sinonasal Malignancies Involving the Frontal Sinus: A Mono-Institutional Experience of 84 Cases and Systematic Literature Review

**DOI:** 10.3390/jcm12093186

**Published:** 2023-04-28

**Authors:** Giorgio Sileo, Marco Valentini, Giacomo Gravante, Giulia Monti, Alberto D. Arosio, Maurizio Bignami, Paolo Battaglia, Paolo Castelnuovo, Mario Turri-Zanoni

**Affiliations:** 1 Department of Otorhinolaryngology Head and Neck Surgery, ASST Sette Laghi, Ospedale di Circolo e Fondazione Macchi, University of Insubria, 21100 Varese, Italy; 2 Department of Otolaryngology Head and Neck Surgery, ASST Lariana, Ospedale Sant’Anna, University of Insubria, 22042 Como, Italy; 3 Head and Neck Surgery and Forensic Dissection Research Center (HNS&FDRc), Department of Biotechnology and Life Sciences, University of Insubria, 21100 Varese, Italy

**Keywords:** endoscopy surgery, skull base, frontal sinus, sinonasal cancer, craniofacial resection, osteoplastic flap, draf procedure, multidisciplinary cancer treatment

## Abstract

Frontal sinus involvement by malignant tumors is a rare finding. Therefore, a systematic literature review along with a personal case series may contribute to defining more accurately the epidemiology, treatment options, and outcomes of these neoplasms. This is a retrospective review of patients affected by frontal sinus malignancies surgically treated in a tertiary-care referral center over a period of 20 years. Moreover, a systematic literature review of studies describing frontal sinus cancers from 2000 to date was performed according to PRISMA guidelines in order to analyze current evidence about the treatment and outcomes of such a rare disease. Our retrospective review was basedon 84 cases, treated with an exclusive endoscopic approach in 43 cases (51.2%), endoscopic approach with frontal osteoplastic flap in 6 cases (7.1%), and transfacial or transcranial approaches in 35 cases (41.7%). The five-year overall, disease-specific, disease-free, and recurrence-free survivals were 54.6%, 62.6%, 33.1%, and 59.1%, respectively. Age, dural involvement, type of surgical resection, and surgical margin status were significantly associated with the survival endpoints. In conclusion, the involvement of the frontal sinus is associated with a poor prognosis. Multidisciplinary management, including specific histology-driven treatments, represents the gold standard for improving outcomes and minimizing morbidity.

## 1. Introduction

Frontal sinus malignancies are uncommon, and they are usually the result of a direct extension of the tumor from the nasal cavity or anterior ethmoid sinuses into the frontal sinus [1]; conversely, malignancies directly arising from the sinus itself constitute an extremely rare finding [2].

The most frequent histologic types are represented by squamous cell carcinomas (SCC, 39.8%), mature B-cell lymphomas (17.5%), epithelial neoplasms not otherwise specified (10.5%), and adenocarcinomas (ADC, 9.9%) [3]. Finally, metastatic disease to the frontal sinus has been reported occasionally in the literature, and, in these cases, the most common primaries are represented by kidney, breast, lung, and gastrointestinal tract tumors [4].

Overall, frontal sinus malignancies show a very poor prognosis, with a 5-year disease-specific survival (DSS) of 44.2% regardless of the histology [1]. Several reasons account for such dismal outcomes, including late diagnosis due to non-specific symptoms, the biology of the disease [5], and the early propensity of tumors involving this area to easily invade the orbit and the anterior cranial fossa, which might be a common finding even at diagnosis. Nonetheless, even considering the rarity of the disease, an update on the current trends in its management as well as a description of the up-to-date literature regarding this specific subset of sinonasal malignancies might help in refining the clinical practice. Therefore, the aim of the present study was to provide updated evidence from the literature regarding the management of frontal sinus malignancies along with the analysis of a cohort of patients affected by such cancers treated in a tertiary care referral center for skull base surgery.

## 2. Materials and Methods

### 2.1. Systematic Literature Review

A systematic literature review regarding the management of malignant tumors involving the frontal sinus was performed according to the Preferred Reporting Items for Systematic Reviews and Meta-Analyses (PRISMA) 2020 guidelines [6]. A database search of PubMed, EMBASE, and the Cochrane Review (from January 2000 to January 2023) was performed to identify suitable articles. Considering the substantial advances in the treatment strategies for sinonasal malignant tumors, studies published before 2000 were excluded.

The PICOS (Participants, Interventions, Comparisons, Outcomes, and Study design) criteria utilized were as follows: Participants (P), patients with a primary malignant tumor of the frontal sinus or with secondary involvement of the sinus; Intervention (I), surgical (either open or transnasal endoscopic approaches) or non-surgical treatment; Comparator (C), observation; Outcomes (O), survivals and recurrence rates; and Study design (S), retrospective and prospective cohort studies. Relevant keywords, phrases, and medical subject headings (MeSH) databases were used according to each database’s requirements. The following is an example of a search strategy used for PubMed/MEDLINE: ((“Nose Neoplasm”[Title/Abstract] OR “Nasal Neoplasm”[Title/Abstract] OR “Neoplasm Nasal”[Title/Abstract] OR “Cancer of Nose”[Title/Abstract] OR “Nose Cancer"[Title/Abstract] OR “Cancer Nose”[Title/Abstract] OR “Nasal Cancer”[Title/Abstract] OR “Cancer Nasal”[Title/Abstract] OR “Cancer of the Nose”[Title/Abstract])) AND ((“Frontal Sinus”[Mesh]) OR ((“Frontal Sinus”[Title/Abstract]) OR (“Sinus Frontal”[Title/Abstract]))) AND (2000:2023[pdat]). The “cited by” function in Google Scholar was used to identify additional articles. The last search was performed on 7 January 2023. Two independent authors (G.S. and M.V.) conducted the electronic search. The following inclusion criteria were considered: full English language text, primary malignant tumors arising from or extending into the frontal sinus, and available individual patient data. Exclusion criteria were as follows: benign and/or borderline sinonasal tumors, a metastatic disease involving the frontal sinus, scientific articles on animals, articles with missing data, database-based review of cases, and full-text not available. After the removal of duplicates, all titles and abstracts were evaluated using the inclusion and exclusion criteria. The full texts of the remaining articles were scrutinized in their entirety to determine final eligibility. At the abstract review stage, we included all studies deemed eligible by at least one author. At the full-text review stage, disagreements were resolved by consensus. The included articles were examined for data extraction, including the number of patients treated, age, sex, site of origin, histology, staging, treatment, and follow-up status.

### 2.2. Mono-Institutional Cohort

The institutional database on cancers of the Otolaryngology–Head and Neck Surgery Department of the University of Insubria-Varese was retrospectively analyzed for patients affected by sinonasal malignancies primarily originating or secondarily involving the frontal sinus, who were treated according to a multimodal strategy including surgery. Only patients treated between January 2000 and January 2022 were included. Demographic data, tumor characteristics, imaging studies, surgical and pathological reports, previous treatments and adjuvant therapy, complications, and follow-up were retrieved. Details regarding diagnosis and the multidisciplinary management protocols of sinonasal malignancies at the present Institution have been extensively described in previous papers [7,8]. In detail, surgery was performed in the case of well-differentiated tumors and poorly-differentiated tumors non-responders to induction chemotherapy. For the evaluation of the response rate to induction chemotherapy, the RECIST 1.1 criteria have been followed [9]. The definition of responders versus non-responders as well as indications for subsequent treatment planning have been described in a previous publication [10]. Regarding the skull base reconstructive plan after surgical resection, local endonasal flaps (e.g., nasoseptal or anterior ethmoidal arteries’ septal flaps) were used whenever feasible and if not involved by cancer. The pericranial flap was harvested in the case of transcranial approaches, whilst pedicled free flaps were generally employed after transfacial resections.

All cases were re-classified according to the 8th edition of the “TNM classification of malignant tumors” for sinonasal cancer [11]. All neoplasms were classified according to the 4th edition of the “WHO classification of Head and Neck tumors” [12]. Written informed consent was obtained from each participant/patient for study participation and data publication. The study was approved by the Institutional Review Board (Insubria Board of Ethics, approval number 0033025/2015). All study procedures were performed in accordance with the 1964 World Medical Association’s Declaration of Helsinki and its later amendments or comparable ethical standards.

### 2.3. Statistical Analysis

The main endpoints analyzed were overall survival (OS), disease-specific survival (DSS), disease-free survival (DFS), and recurrence-free survival (RFS). OS was defined as the time from surgery to the last follow-up or death from any cause. DSS was defined as the time interval between surgery and death from disease. DFS was defined as the time from surgery to the first relapse at any site or death from any cause. RFS was defined as the time from surgery until relapse (either local, regional, or distant). Survival probability was assessed using the Kaplan–Meier survival analysis and the log-rank test was performed to compare survivals. Age (i.e., ≤60 years vs. >60 years), presentation (i.e., naive vs. relapses), histology, pT classification (i.e., pT3 vs. pT4a-4b), tumor epicenter (i.e., frontal vs. others), dural involvement (i.e., yes vs. no), type of surgery (i.e., exclusive endoscopic vs. combined vs. external approaches), surgical margins (i.e., R0 vs. R+), grading (i.e., G1–2 vs. G3), and adjuvant treatment (i.e., yes vs. no) were tested as prognostic factors.

A multivariate proportional hazard Cox regression was used for the same endpoints (OS, DSS, DFS, and RFS) considering variables significant in univariate analysis and/or with relevant clinical value. The results are presented in terms of hazard ratios (HR), 95% confidence intervals (CIs), and *p*-values. All statistical tests were two-tailed and statistical significance was considered when *p*-value ≤ 0.05. IBM SPSS software^®^, version 25 (Chicago, IL, USA), was used to perform all statistical analyses.

## 3. Results

### 3.1. Systematic Literature Review

**Figure 1:**
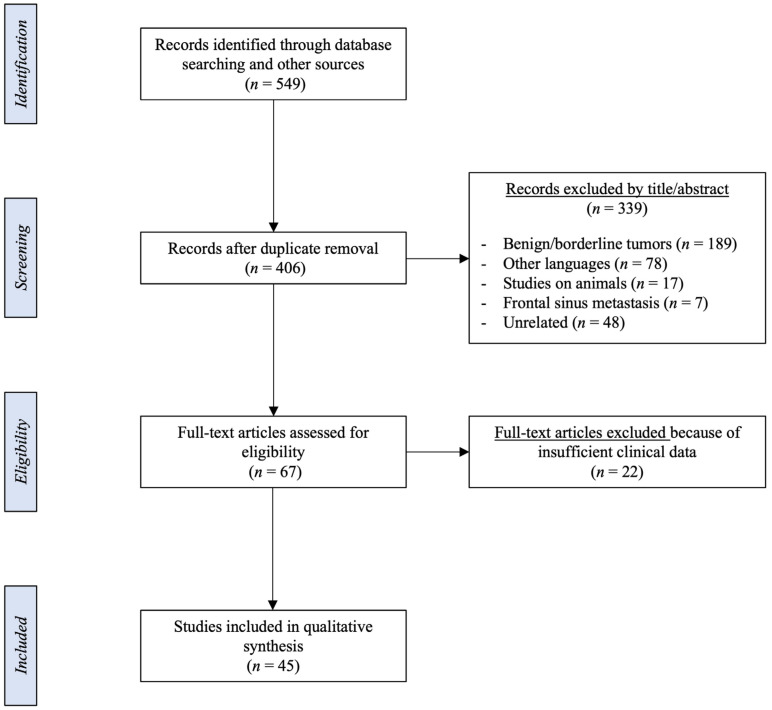
PRISMA flowchart summarizing the methods used in the present study.

The PRISMA flow diagram is shown in Figure 1. After duplicate removal, 406 potentially relevant records were identified through database searching and other sources. Of these, 339 studies were excluded after the title and abstract review, while the full texts of the remaining 67 articles were examined for further review. Finally, 45 studies were screened for the aforementioned eligibility criteria and included in the systematic review.

A total of 51 cases were described in the selected studies (Table 1). Globally, 19 patients (37.3%) were female and 32 were male (62.7%); the mean age was 54 years (range 11–84 years). In 44 cases (86.3%) the tumor origin was at the level of the frontal sinus. Regarding tumor histology, a great variability was observed: sarcoma represented the most frequent histotype (17 cases, 33.3%), followed by SCC (11 cases, 21.6%), lymphatic tumors (9 cases, 17.6%), ADC (3 cases, 5.9%), neuroectodermal tumors (5 cases, 9.8%), salivary gland cancers (SGC, 2 cases, 3.9%), mucosal melanomas (MM, 2 cases, 3.9%), NUT carcinomas (1 case, 2%), and malignant solitary fibrous tumor (1 case, 2%). Regarding the management strategy, lymphatic tumors were managed with non-surgical treatment (radiotherapy, RT; chemotherapy, CHT; or chemoradiotherapy, CRT) in all cases. Among the 33 cases surgically treated, 15.1% (5/33) underwent endoscopic endonasal resection (EER), 66.7% (22/33) craniofacial resection (CFR), and 18.2% (6/33) cranio-endoscopic resection (CER). Induction chemotherapy (iCHT) was reported in 5,8% of cases (3 cases: one case each of SNUC, Ewing sarcoma, and rhabdomyosarcoma, respectively), followed by further treatments based on the degree of tumor response. Post-treatment complications were observed in 11 cases (20.4%), with minor complications occurring in 5 cases (45.5%) and major ones in 6 cases (54.5%) (Table 1).

**Table 1:** Summary of the analyzed case series describing treatment and outcomes of sinonasal malignancies involving the frontal sinus.

Author	Year	N° of Cases	Age	Gender	Origin	Histology	T	N	M	Management	Complications	Follow-Up (Months)	Status	Recurrence (Where)	Months from Treatment	Recurrence Treatment
Abrahao [13]	2000	1	59	F	FS	ADC	4b	NA	NA	CFR + CHRT	Cranioplasty infection	10	AWD	Nasal cavity	10	NA
Rodrigo [14]	2000	1	53	M	FS	Malignant fibrous histiocytoma	NA	NA	NA	CFR	-	68	AWD	FS + lung	60	CHT
Gallia [15]	2005	1	44	M	FS	Synovial sarcoma	4b	0	0	CFR + CHRT	-	2	NED	-	-	-
Yoshida [16]	2006	1	74	M	FS	SCC	4b	0	0	CFR + RT	-	20	DOD	FS + lung	3	CFR + CHRT
Nemet [17]	2006	1	84	M	FS	B-cell lymphoma	IE	-	-	RT	-	9	DOD	-	-	-
Ogunleye [18]	2006	1	31	F	FS	MPNST	4b	0	0	CFR + RT	-	24	NED	-	-	-
Gerlinger [19]	2007	1	60	M	FS	SNUC	4a	0	0	CFR + RT	-	12	NED	-	-	-
Chain [20]	2007	1	55	M	FS	B-cell lymphoma	IIE	-	-	CHRT	-	18	NED	-	-	-
Chu [21]	2008	1	61	M	FS	SNEC	4a	0	0	ER	-	14	NED	-	-	-
Ichinose [22]	2009	1	66	M	FS	SCC	4b	0	0	CFR	-	48	NED	-	-	-
Gray [23]	2009	1	17	M	ES	Ewing sarcoma	4a	0	0	IMPT + CHT	Frontal mucocele	7	NED	-	-	-
Lazzeri [24]	2010	1	78	M	ES	MM	4b	NA	NA	ER + RT	-	1	DOD	-	-	-
Ito [25]	2010	2	43 (mean)	F	FS	Osteosarcoma	4b	X	X	Best supportive care CER	-	3 7	DOD DOD	-	-	-
Kim [26]	2011	3	69 (mean)	M	FS	SCC on IP	4b 4a 4a	0 2b 0	0	CFR + RT RT CHT/RT	-	3 5 23	lost DOD DOD	-	-	-
Madana [27]	2011	1	48	F	FS	SCC	4a	0	0	CFR + RT	-	12	NED	-	-	-
Wadhera [4]	2011	1	53	M	FS	ADC	4b	0	0	CHRT	-	12	AWD	-	-	-
Bercin [28]	2011	1	47	F	ES	Fibrosarcoma	4b	0	0	CER	-	24	NED	-	-	-
Soyka [29]	2011	1	43	F	FS	Malignant solitary fibrous tumor	4a/4b	NA	NA	CER + RT	Osteomyelitis	36	NED	-	-	-
Kim [30]	2011	1	42	M	FS	B-cell lymphoma	IVE	-	-	CHT	-	50	NED	-	-	-
Hosokawa [31]	2012	1	71	M	FS	SCC	4b	0	0	CER	-	40	NED	-	-	-
Fukumitsu [32]	2012	1	71	M	FS	SCC	4b	X	X	IMPT	Brain necrosis	22	DOC	-	-	-
Peregud-Pogorzelski [33]	2012	1	11	F	FS	Rhabdomyosarcoma	4a/4b	1	0	iCHT + ER + CHRT	Mycotic sinusitis	1	NED	-	-	-
Zhang [34]	2014	1	66	M	FS	SCC	4b	0	0	CFR + RT	-	6	NED	-	-	-
Lee [35]	2014	1	74	M	ES	MPNST	4a	0	0	CFR	Skin flap necrosis	6	NED	-	-	-
Jankowski [36]	2014	2	39.5 (mean)	M	ES	ONB	B C	0 N1	0	ER CHT + CFR + RT	-	6 24	NED NED	-	-	-
Verim [37]	2014	1	69	F	FS	Plasmacytoma	4b	0	0	CFR + RT	-	18	NED	-	-	-
Tomovic [38]	2014	1	21	F	FS	Angiosarcoma	4b	1	1	CHRT	-	12	NED	-	-	-
Khan [39]	2015	1	69	M	FS	B-cell lymphoma	IE	-	-	CFR + CHT	-	36	NED	-	-	-
Kieliszak [40]	2015	1	63	M	FS	Granulocytic sarcoma	4a	0	0	CFR + CHT	Neutropenic fever	1	DOC	-	-	-
Kuan [41]	2017	1	75	M	FS	SCC on IP	4a	0	0	CHT + ER	-	12	NED	-	-	-
Minato [42]	2017	1	66	F	FS	NUT carcinoma	4b	0	0	CHT + RT		13	DOD	Bone + liver	NA	CHT
Cannon [43]	2017	3	69 (mean)	F	FS	Biphenotypic sarcoma	4a 4b 4b	0	0	ERTC CFR + CER Awaiting surgery	-	17 NA NA	NED NED AWD	ACF - -	17 - -	Revision ERTC - -
Sienna [44]	2018	1	80	M	ES	SNUC	4a	2	0	iCHT + CHRT	-	17	DOD	FS + brain	4	CHRT
Kim [45]	2018	1	63	M	FS	ITAC	4a	0	0	CFR	-	6	NED	-	-	-
Nagafuji [46]	2018	1	67	M	FS	B-cell lymphoma	IIE	-	-	CHT	-	12	NED	-	-	-
Biswas [47]	2019	1	21	F	FS	ALCL	IE	-	-	CHRT	Cutaneous nasal fistula	40	NED	-	-	-
Tirelli [48]	2019	1	45	M	FS	ACC	4a	0	0	CFR	Diplopia	24	NED	-	-	-
Alvi [49]	2019	1	44	F	FS	Biphenotypic sarcoma	4a	0	0	CFR + RT	-	360	NED	FS + ES	350	CFR
Knudson [50]	2019	1	11	M	FS	B-cell lymphoma	IIE	-	-	CHT	Brain herniation	12	NED	-	-	-
Lee [51]	2020	1	53	M	FS	SCC	4a	0	0	CER + CHRT	-	36	NED	-	-	-
Esteves Costa [52]	2020	1	12	F	FS	Ewing sarcoma	4a	0	0	iCHT + RT	-	17	NED	-	-	-
Baudoin [53]	2021	1	47	M	MS	ACC	4a	0	0	CFR + RT	-	NA	NED	FS	NA	CFR
Andron [54]	2021	1	75	F	FS	Plasmacytoma	4a	0	0	CFR + RT	-	22	NED	-	-	-
Yoon [55]	2021	1	46	M	FS	B-cell lymphoma	IIE	-	-	CHT	Sepsis	4	DOC	-	-	-
Kieu [56]	2021	1	65	F	FS	MM	4b	0	0	CFR + RT + immunotherapy	-	3	NED	-	-	-

Abbreviations: ACC: adenoid cystic carcinoma; ACF, anterior cranial fossa; ADC, adenocarcinoma; ALCL, ALK-positive anaplastic large cell lymphoma; AWD, alive with disease; CER, cranio-endoscopic resection; CFR, craniofacial resection; CHRT, chemoradiotherapy; DOC, death from other causes; DOD, death from disease; ES, ethmoid sinus; ER, endoscopic resection; FS, frontal sinus; iCHT, induction chemotherapy; IMPT, intensity-modulated proton therapy; IP, inverted papilloma; ITAC, intestinal-type adenocarcinoma; MM, mucosal melanoma; MPNST, malignant peripheral nerve sheath tumor; MS, maxillary sinus; NA, not available; NED, no evidence of disease; ONB, olfactory neuroblastoma; RT, radiotherapy; SCC, squamocellular carcinoma; SNEC, sinonasal neuroendocrine carcinoma; SNUC, sinonasal undifferentiated carcinoma.

The mean follow-up period was 24.7 months (range 1–360 months) and one patient was lost during the follow-up. Recurrences were observed in 8 cases (15.7%), with a mean time to recurrence of 85.4 months. More specifically, relapses were divided as follows: local failure in eight cases and distant metastasis in four cases. At the time of the last follow-up, 34 patients (66.7%) had no evidence of disease (NED); 4 patients (7.8%) were alive with disease (AWD); 9 patients (17.6%) died of disease (DOD) and 3 patients (5.9%) died from other causes (DOC).

The five-year OS, DSS, and DFS were 61.3%, 71.4%, and 41.9%, respectively. After excluding the lymphatic tumors from the survival analysis, the OS, DSS, and DFS were lower: 56.5%, 67%, and 38.9%, respectively.

### 3.2. Mono-Institutional Cohort

During the time span considered, a total of 84 patients were included in the study. In 24 cases (28.6%) the malignant tumor originated within the frontal sinus, whereas in the remaining 60 cases (71.4%) a secondary involvement of the sinus was reported. There was a slight predominance of males (male-to-female ratio of 1.3:1) and the mean age of the population was 60 years (range, 17–83 years; median, 62.5 years). Most patients were naive at presentation (56 cases, 66.7%) whilst 28 patients (33.3%) were treated for relapse or persistence of disease. The most frequent histology types encountered were squamous cell carcinoma (SCC) in 21 cases (25%), mucosal melanoma (MM) in 14 cases (16.7%), and poorly-differentiated carcinomas (neuroendocrine carcinomas, NEC, or sinonasal undifferentiated carcinomas, SNUC) in 13 cases (15.5%). Regarding the surgical resection, endoscopic endonasal resection (EER) was performed in 20 cases (23.8%), EER with frontal osteoplastic flap (OPF) in 6 cases (7.1%), endoscopic resection with transnasal craniectomy (ERTC) in 23 cases (27.3%), combined cranio-endoscopic resection (CER) in 23 cases (27.3%), and craniofacial resection (CFR) in 12 cases (14.2%). Table 2 summarizes the clinicopathological characteristics of the study population. As for frontal sinus involvement, the staging was at least pT4a in all cases, with the exception of five patients affected by MM staged as pT3. A total of 16 patients (19%) experienced postoperative complications: cerebrospinal fluid (CSF) leak (4 cases, 25%), free flap necrosis (3 cases, 18.7%), pneumocephalus (2 cases, 12.5%), extradural abscess (2 cases, 12.5%), donor site morbidity, pneumonia, deep vein thrombosis, epistaxis, and sepsis (1 case each, 6.2%). Most of the patients received adjuvant treatment (61 cases, 72.6%) whereas 23 patients did not, as it was not indicated after multidisciplinary discussion (e.g., salvage surgery after chemo-radiotherapy failure). After a mean follow-up of 39 months (range, 6–149 months), 33 patients (39.3%) were alive without evidence of disease (Table 2).

**Figure 2:**
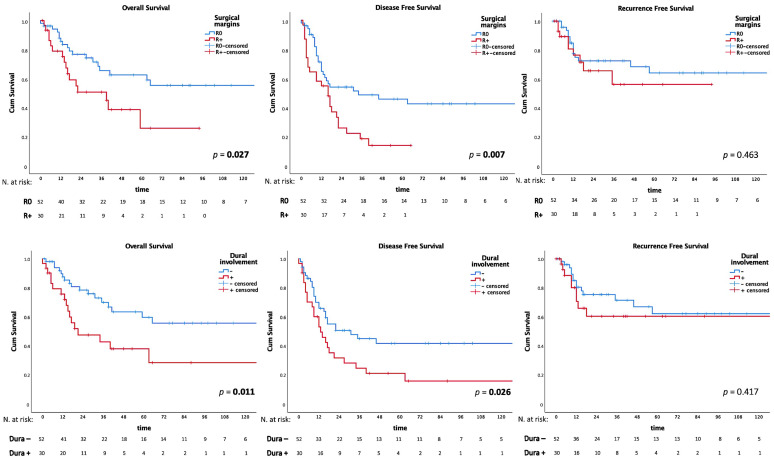
Univariate analysis: Kaplan–Meier curves for overall survival (OS), disease-free survival (DFS), and recurrence-free survival (RFS) according to the status of surgical margins and dural involvement. Bold values are statistically significant.

The five-year OS, DSS, DFS, and RFS were 54.6%, 62.6%, 33.1%, and 59.1%, respectively. Age, dural involvement, type of surgical resection, and surgical margin status were significantly associated with the survival endpoints on univariate analysis (Table 3, Figure 2). It is noteworthy that histology was associated with statistically significant differences only in terms of DFS (*p* = 0.049), with MM and salivary gland cancers reaching the lowest survival rates (two-year DFS of 20% and 18.5%, respectively). After excluding lymphatic tumors from the survival analyses, the five-year OS, DSS, DFS, and RFS were 50.8%, 58.9%, 33.3%, and 60.2%, respectively.

No statistically significant differences in survival considering the variable “tumor epicenter” (frontal sinus vs. other sinuses) were found.

The total recurrence rate was 39.3% (33/84 patients, Table 2) and half of the patients experienced early recurrences, within 9 months. Local failure was the most frequent event in 19/33 cases (57.6%), while dissemination of disease was observed in 13/33 cases (39.4%): multi-organ (7 cases), brain (3 cases), liver (1 case), and bones (2 cases). Treatment of recurrences was surgically-based in 7/33 cases (21.2%), non-surgical (RT and/or CRT) in 19/33 cases (57.6%), and 7/33 (21.2%) patients were addressed to best supportive care. Age was confirmed to be an independent prognostic factor in terms of OS and DSS in multivariate analysis (Table 4). The type of surgical resection was associated with prognosis since patients affected by locally advanced cancers who required more extensive procedures (e.g., transcranial approaches) were more prone to recurrences (HR 3.3 in RFS, *p* = 0.006), while the status of surgical margins was associated with a close-to-significance increased risk of death and recurrence (HR 1.7 in DFS, *p* = 0.062).

## 4. Discussion

Frontal sinus malignancies are rare, comprising less than 2% of all cases of sinonasal malignancy [57], and include both tumors arising from the sinus itself and those extending into it from adjacent anatomical areas [3]. Therefore, the literature on studies focusing on malignancies involving this specific anatomical region is scarce and it is mainly represented by single case reports, with wide heterogeneity in histologies and treatment strategies. The work by Bhojwani et al. [1] reports 171 cases of malignancies involving the frontal sinus treated from 1973 to 2012 and represents the largest reported cohort currently available in the literature. Being a database-based study, it collects data derived from different institutions and it considers a broad period of time, for the most part before the introduction of recent advances in multimodal treatment strategies [58]. All this should be taken into consideration when drawing conclusions for clinical practice. Nonetheless, Bhojwani et al. report that frontal sinus malignancies are characterized by a very unfortunate prognosis, with a five-year DSS of 44.2% [1]. The present literature review seems to confirm this trend, considering that the pooled five-year DSS of all the included case reports was 67% after excluding lymphatic tumors, which are managed with (chemo)radiation and often characterized by better prognosis. Epithelium-derived cancers, on the contrary, are advanced by definition and pose challenges for both diagnosis and treatment, which significantly impacts prognosis. Common manifestations include swelling of the forehead, frontal headache, and indirect signs of orbital invasions, such as diplopia and proptosis [59]. Unfortunately, the symptoms are generally associated with an extension of the disease beyond frontal sinus boundaries, with the involvement of adjacent areas such as the orbit or the intracranial compartment, which is not infrequent at the time of diagnosis [2].

In light of the fact that frontal sinus cancers are rare, this sinus is not considered a “primary site” in the staging system promoted by the American Joint Committee on Cancer (AJCC) [60] and the Union for International Cancer Control (UICC) [11]: these organizations, in fact, primarily mentioned the nasal cavity, maxillary, and ethmoid sinuses and consider the frontal sinus only in the cases of secondary involvement (stage T4a). In 2002, the University of Florida grouped patients with frontal sinus malignancies into three stages: stage I, if limited to the site of origin; stage II, if the tumor extends to adjacent sites (i.e., orbit, nasopharynx, paranasal sinuses, skin, and pterygomaxillary fossa); and stage III, in the case of skull base, pterygoid plate, or intracranial involvement [61].

**Figure 3:**
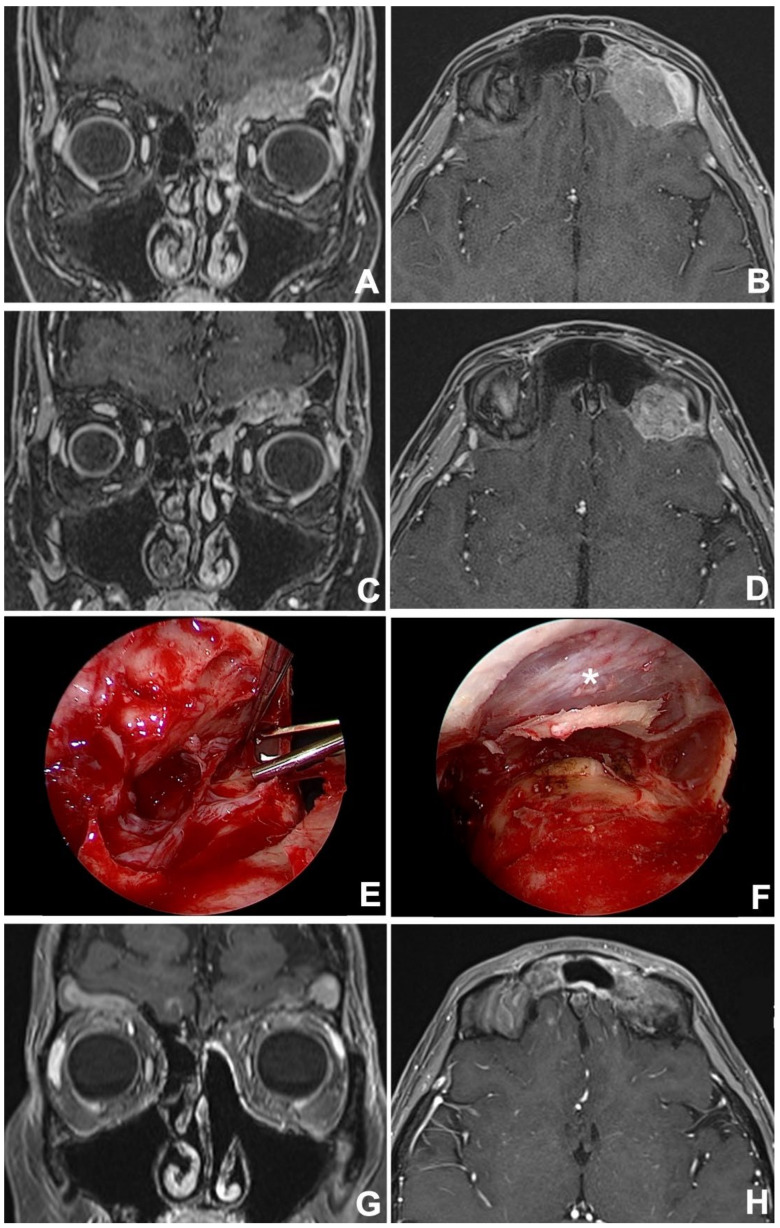
Contrast-enhanced MRI in coronal (**A**) and sagittal (**B**) views showing a left frontoethmoidal INI-1 deficient squamous cell carcinoma. After 3 cycles of induction chemotherapy (TPF regimen) an MRI in the coronal (**C**) and axial (**D**) view revealed an incomplete response. Tumor persistence was resected via a combined approach: endoscopic transnasal and osteoplastic flap (**E**), with an exposition of anterior cranial fossa dura (*) which appeared to be free from tumor infiltration (**F**). At 12 months, postoperative contrast-enhanced MRI in coronal (**G**) and axial (**H**) views revealed no local recurrence.

Diagnosis is made on imaging studies such as computed tomography (CT) and magnetic resonance (MR), which should precede biopsy, which is mainly performed via a minimally invasive approach (i.e., endoscopic transnasal approach), in order to define the histological profile of the cancer before planning the definitive treatment. This should be recommended in clinical practice since nowadays histotype has proven to be one of the main determinants of prognosis and essential to establishing the most appropriate multidisciplinary treatment strategy [5,7,8]. In this regard, the slightly better oncologic outcomes observed in the present cohort, compared to the observation by Bhojwani et al. [1], with a five-year DSS of 62.6%, might be in part explained by the implementation of histology-driven multimodal strategies in the management of such cancers, especially in locally-advanced and poorly-differentiated neoplasms (Figure 3).

In the present case series, the most common histotype was SCC (25%), in line with the literature [1,2], followed by aggressive entities such as MM (16.7%), SNEC, and SNUC (15.5%). The last three were much more common than what was observed in the systematic literature review (MM, 3.9%, and neuroectodermal tumors, 9.8%). Indeed in the database analysis by Bhojwani et al. [1] melanomas and neuroepitheliomatous neoplasms accounted for less than 6% of all frontal sinus malignancies. In one large recently published series on endoscopically treated sinonasal malignant tumors [7], the percentages of MM and SNEC/SNUC were 7.6–9.6% and 6.1–8.6% respectively, thus stressing the difference in histotype distribution between “primary sites” (ethmoid, nasal cavity, and maxillary sinus) and frontal sinus cancers, in which undifferentiated and aggressive histotypes are significantly represented, as shown by the present study.

**Figure 4:**
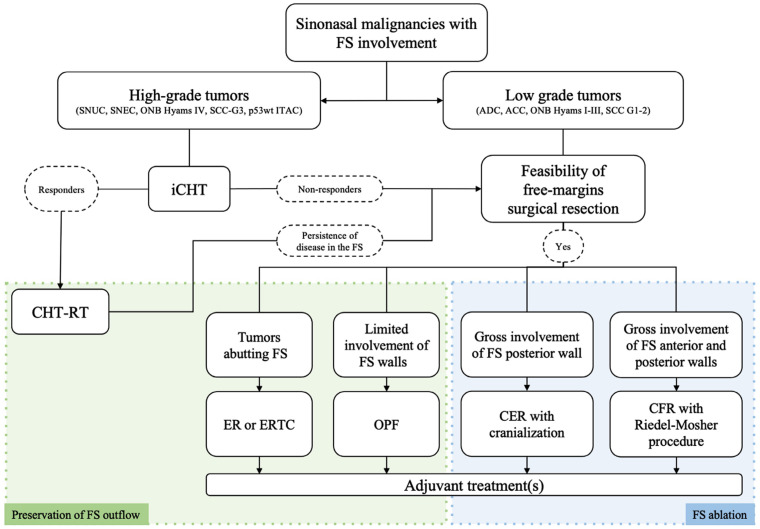
Flowchart of the study protocol, describing the multimodal treatment algorithm for the management of the malignancies involving the frontal sinus. The response rate to induction chemotherapy was defined according to the RECIST 1.1 criteria [9]. Abbreviations: ACC, adenoid cystic carcinoma; ADC, adenocarcinoma; BSC, best supportive care; CER, cranio-endoscopic resection; CFR, craniofacial resection; CHT, chemotherapy; EER, endoscopic endonasal resection; ERTC, endoscopic resection via transnasal craniectomy; FS, frontal sinus; iCHT, induction chemotherapy; ITAC, intestinal-type adenocarcinoma; OPF, osteoplastic flap; RT, radiotherapy; SCC, squamous cell carcinoma; SNEC, sinonasal neuroendocrine carcinoma; SNUC, sinonasal undifferentiated carcinoma.

**Figure 5:**
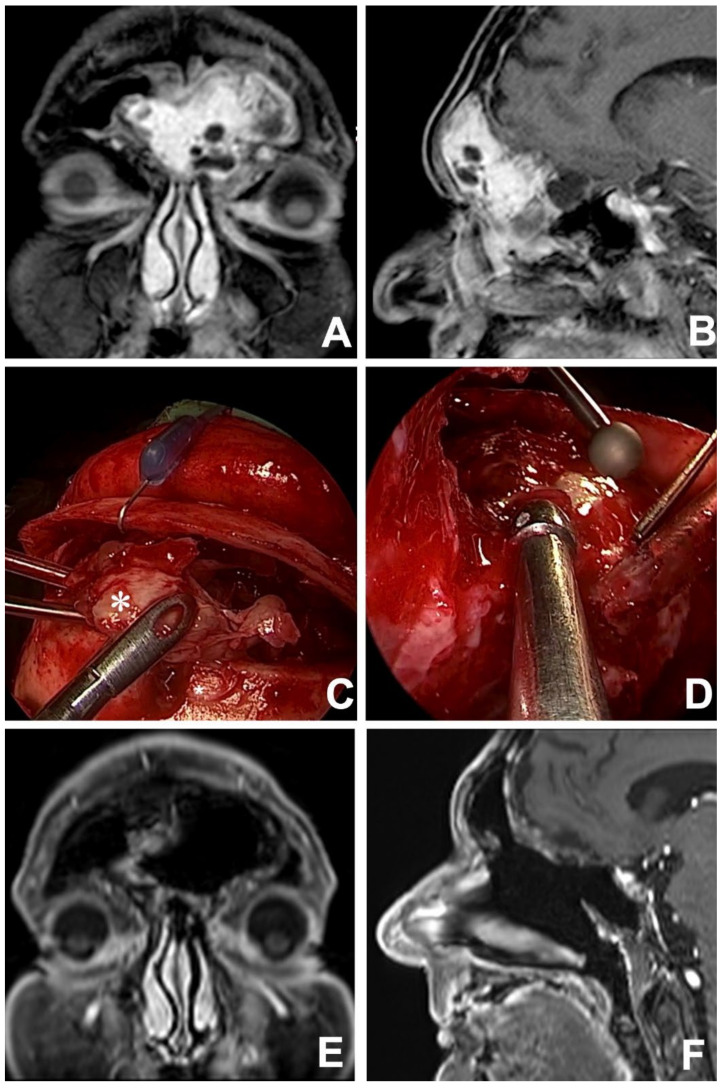
Preoperative MRI in coronal (**A**) and sagittal (**B**) views showing a frontoethmoidal neoplasm with massive bilateral frontal sinus involvement. After an endoscopic transnasal biopsy, the lesion was histologically defined as biphenotypic sinonasal sarcoma. The lesion (*) was resected via a combined surgical approach: frontal sinus osteoplastic flap (**C**) and endoscopic transnasal approach (**D**), achieving free resection margins. A contrast-enhanced coronal (**E**) and sagittal (**F**) MRI performed 2 years after treatment proved no evidence of residual disease and ruled out local recurrences.

Among rhinologists, the frontal sinus is recognized to be one the most challenging areas to reach surgically and, even nowadays, external approaches might be needed to manage specific situations (Figure 4) [62]. Over the years, instrumental and technological evolutions have allowed the application of minimally-invasive endoscopic approaches in the management of frontal disease, even in the case of malignant tumors. Currently, endoscopic transnasal and external approaches (i.e., transcranial/transfacial) should be considered as complementary techniques that must be mastered by the skull base surgeon, who must be able to switch from endonasal to external procedures whenever required, depending on intraoperative findings (Figure 5).

Undoubtedly, the goal of surgery is to achieve a free-margin resection, as the status of surgical margins has proven to be a transversal negative prognosticator among sinonasal malignancies [8], strictly linked with the high rate of relapses. The oncological concept of free-margin resection was supported also by our cohort, in which infiltrated surgical margins were associated with a significantly increased hazard ratio (HR) in 5-year DFS on multivariate analysis (*p* = 0.062).

The present study has some limitations that cannot be neglected. Firstly, it is based on a retrospective analysis of cases over a 20-year period, which might have introduced biases related to changes in staging systems and treatment modalities. Second, the wide heterogeneity of histologies within the broad panorama of sinonasal malignancies forced us to use a simplistic stratification in a few groups. Third, our systematic literature review is mainly represented by single case reports, hence with low scientific evidence, and heterogeneity in histotypes and treatment strategies. It should be mentioned that there might be larger studies in the literature focusing on sinonasal malignancies, with some reported cases of frontal sinus involvement. However, it was not possible to extrapolate from these studies individual data of patients with frontal sinus involvement adequate for the present analysis and therefore they were excluded from this systematic literature review.

Considering all these study limitations and open issues, it appears clear that the frontal sinus still represents a critical area in the management of sinonasal malignancies, with difficulties that have not been completely overcome. Future efforts should be devoted to focusing on prevention and early diagnosis as well as refining the multimodal histology-driven treatment strategies, which in the future will be tailored to the specific genetic and molecular assets of each patient, limiting external destructive surgical approaches to properly selected cases.

## 5. Conclusions

Malignant tumors involving the frontal sinus are rare and typically present as a secondary involvement of ethmoidal malignancies. Given the proximity to adjacent noble structures, such as anterior cranial fossa and orbit, they are usually diagnosed in advanced stages and associated with poor prognosis. Factors significantly associated with the survival endpoints were age, dural involvement, type of surgical resection, surgical margin status, and histology. Hence, nowadays, multimodal treatment protocols should be histology-based and patient-tailored, in order to maximize the chances of cure and minimize patient morbidity.

## Figures and Tables

**Table 2:** Demographic and clinical data of the mono-institutional case series.

Variable		*n*	%
Age (years)	Mean	60	/
	Median	62	/
	Range	17–83	/
Gender	Male	48	57.1%
	Female	36	42.9%
Presentation	Naive	56	66.7%
	Relapse/persistence	28	33.3%
Origin	Frontal sinus	24	28.6%
	Other sinuses	60	71.4%
T classification	T3	5	6.0%
	T4a	30	35.7%
	T4b	49	58.3%
N classification	N0	82	97.6%
	N+	2	2.4%
M classification	M0	81	96.4%
	M1	3	3.6%
Histotype	Carcinoma	21	25.0%
	Mucosal melanoma	14	16.7%
	SNEC/SNUC	13	15.5%
	Adenocarcinoma	12	14.3%
	ITAC	9	75.0%
	non-ITAC	2	16.7%
	NOS	1	8.3%
	Soft tissue tumors	10	11.9%
	ONB	7	8.3%
	Salivary gland cancer	5	5.9%
	ACC	3	60.0%
	Acinic cell	1	20.0%
	Salivary	1	20.0%
	Lymphatic tumors	2	2.4%
Surgical treatment	EER/ERTC	43	51.2%
	EER + OPF	6	7.1%
	CER/CFR	35	41.7%
Dural resection	Yes	52	61.9%
	No	32	38.1%
Margins status	R0	53	63.1%
	R1	31	36.9%
Adjuvant treatments	Yes	61	72.6%
	No	23	27.4%
Complications	Yes	16	19.0%
	No	68	81.0%
Follow-up	Mean	39	/
	Range	6–149	/
Recurrence	Yes	33	39.3%
	No	51	60.7%
Status	NED	33	39.3%
	AWD	16	19.0%
	DOC	7	8.3%
	DOD	28	33.4%

Abbreviations: ACC, adenoid cystic carcinoma; AWD, alive with disease; CER, cranio-endoscopic resection; CFR, craniofacial resection; DOC, death from other causes; DOD, death from disease; EER, endoscopic endonasal resection; ERTC, endoscopic resection via transnasal craniectomy; ITAC, intestinal-type adenocarcinoma; NED, no evidence of disease; NOS, not otherwise specified; ONB, olfactory neuroblastoma; OPF, osteoplastic flap; SCC, squamocellular carcinoma; SNEC, sinonasal neuroendocrine carcinoma; SNUC, sinonasal undifferentiated carcinoma.

**Table 3:** Univariate analysis of overall survival, disease-specific survival, disease-free survival, and recurrence-free survival according to prognostic factors.

Variable		OS			DSS			DFS			RFS		
		3-y (%)	5-y (%)	*p*-Value	3-y (%)	5-y (%)	*p*-Value	3-y (%)	5-y (%)	*p*-Value	3-y (%)	5-y (%)	*p*-Value
Age	≤60 years	76.2 ± 8.2	71.1 ± 9.1	**0.005**	82.6 ± 7.4	82.6 ± 7.4	**0.003**	46.6 ± 8.8	46.6 ± 8.8	0.163	72.9 ± 8.3	65.6 ± 10.2	0.522
	>60 years	49.2 ± 7.9	38.7 ± 8.2		56.6 ± 7.8	44.6 ± 8.7		30.7 ± 7.2	25.1 ± 6.9		63.5 ± 8.6	57.7 ± 9.6	
Presentation	Naive	56.5 ± 7.4	49.7 ± 7.9	0.469	65.4 ± 6.9	57.5 ± 8.0	0.566	39.7 ± 7.1	36.8 ± 7.1	0.658	80.3 ± 6.3	80.3 ± 6.3	**<0.0005**
	Relapse	67.9 ± 10.2	56.0 ± 11.4		71.9 ± 10.0	64.7 ± 11.3		32.6 ± 9.4	28.5 ± 9.1		45.3 ± 10.5	31.7 ± 11.1	
pT	pT3	50.0 ± 35.4	50.0 ± 35.4	0.612	50.0 ± 35.4	50.0 ± 35.4	0.810	21.4 ± 18.8	NA	0.290	50.0 ± 25.0	NA	0.112
	pT4a-4b	59.8 ± 6.1	51.0 ± 6.6		66.4 ± 5.8	59.4 ± 6.7		38.4 ± 5.9	36.5 ± 5.9		68.8 ± 6.1	65.0 ± 6.9	
Tumor epicenter	Frontal	68.5 ± 11.1	68.5 ± 11.1	0.097	73.0 ± 10.9	73.0 ± 10.9	0.164	43.9 ± 10.4	36.5 ± 10.9	0.678	62.2 ± 10.6	42.7 ± 13.7	0.146
	Other	56.6 ± 7.1	45.7 ± 7.6		64.5 ± 6.8	54.5 ± 7.9		35.1 ± 6.7	32.9 ± 6.6		69.7 ± 7.4	69.7 ± 7.4	
Histology	SGC	53.3 ± 24.8	NA	0.206	66.7 ± 27.2	NA	0.232	NA	NA	**0.049**	NA	NA	0.347
	MM	39.2 ± 16.4	39.2 ± 16.4		39.2 ± 16.4	39.2 ± 16.4		18.5 ± 11.6	9.3 ± 8.8		80.0 ± 12.6	53.3 ± 23.3	
	SNEC/SNUC	34.6 ± 15.1	34.6 ± 15.1		34.6 ± 15.1	34.6 ± 15.1		28.0 ± 13.6	28.0 ± 13.6		77.9 ± 14.1	77.9 ± 14.1	
	ADC	54.7 ± 15.4	32.8 ± 15.1		72.9 ± 13.5	54.7 ± 18.8		22.2 ± 12.8	22.2 ± 12.8		51.6 ± 15.8	51.6 ± 15.8	
	SCC	72.2 ± 10.7	64.2 ± 12.1		78.2 ± 9.7	69.5 ± 11.9		40.7 ± 11.7	40.7 ± 11.7		60.0 ± 12.0	60.0 ± 12.0	
	Lymphatic tumor	100	100		100	100		50.0 ± 35.4	50.0 ± 35.4		50.0 ± 35.4	50.0 ± 35.4	
	Soft tissue tumor	80.0 ± 12.6	80.0 ± 12.6		80.0 ± 12.6	80.0 ± 12.6		70.0 ± 14.5	70.0 ± 14.5		77.8 ± 13.9	77.8 ± 13.9	
	ONB	80.0 ± 17.9	60.0 ± 21.9		100	75.0 ± 21.7		80.0 ± 17.9	60.0 ± 21.9		100	50.0 ± 35.4	
Dural involvement	yes	42.9 ± 10.0	38.1 ± 9.9	**0.011**	53.7 ± 9.9	47.8 ± 10.5	**0.045**	24.8 ± 8.1	21.3 ± 7.7	**0.026**	60.5 ± 10.5	60.5 ± 10.5	0.417
	no	70.0 ± 7.1	59.7 ± 8.2		74.9 ± 6.7	66.9 ± 8.0		45.1 ± 7.4	41.9 ± 7.6		71.5 ± 7.3	62.2 ± 8.8	
Surgery	EER	61.2 ± 8.7	53.6 ± 9.1	0.093	66.3 ± 8.3	58.9 ± 9.2	0.231	37.9 ± 8.0	30.8 ± 7.9	**0.023**	79.8 ± 6.9	71.8 ± 9.8	**0.009**
	EER/OPF	100	100		100	100		100	100		100	100	
	CER/CFR	51.9 ± 9.1	42.6 ± 9.1		60.9 ± 8.9	54.1 ± 10.2		27.1 ± 7.9	27.1 ± 7.9		47.1 ± 10.1	47.1 ± 10.1	
Surgical Margins	R0	65.5 ± 7.4	62.5 ± 7.7	**0.027**	74.5 ± 6.8	71.1 ± 7.3	**0.015**	48.6 ± 7.5	45.8 ± 7.6	**0.007**	72.7 ± 6.8	64.3 ± 8.2	0.436
	R1/2	50.7 ± 9.9	25.7 ± 12.7		54.6 ± 9.9	31.2 ± 14.7		18.5 ± 7.3	13.8 ± 6.8		56.4 ± 12.3	56.4 ± 12.3	
Grading	G1–2	76.1 ± 9.6	56.4 ± 12.1	0.287	90.9 ± 6.1	67.3 ± 12.6	0.130	54.7 ± 10.8	49.2 ± 11.0	0.104	76.8 ± 9.1	67.2 ± 12.0	0.369
	G3	51.2 ± 9.8	46.6 ± 10.0		59.2 ± 9.6	59.2 ± 9.6		29.1 ± 8.7	29.1 ± 8.7		60.7 ± 10.0	60.7 ± 10.0	
Adjuvant treatment	yes	60.4 ± 7.0	53.7 ± 7.7	0.902	67.1 ± 6.6	59.6 ± 7.8	0.818	39.4 ± 6.7	34.1 ± 6.7	0.820	69.6 ± 6.7	64.7 ± 7.8	0.519
	no	59.9 ± 11.3	47.2 ± 12.0		68.3 ± 11.0	59.8 ± 12.5		33.0 ± 10.6	33.0 ± 10.6		62.6 ± 12.6	52.2 ± 14.2	

Abbreviations: ADC, adenocarcinoma; CER, cranio-endoscopic resection; CFR, craniofacial resection; DFS, disease-free survival; DSS, disease-specific survival; EER, endoscopic endonasal resection; MM, mucosal melanoma; NA, not available; ONB, olfactory neuroblastoma; OPF, osteoplastic flap; OS, overall survival; RFS, recurrence-free survival; SCC, squamocellular carcinoma; SGC, salivary gland cancers; SNEC, sinonasal neuroendocrine carcinoma; SNUC, sinonasal undifferentiated carcinoma. Bold values are statistically significant.

**Table 4:** Multivariate analysis of overall survival, disease-specific survival, disease-free survival, and recurrence-free survival.

Variable	OS			DSS			DFS			RFS		
	HR	HR CI 95%	*p*-Value	HR	HR CI 95%	*p*-Value	HR	HR CI 95%	*p*-Value	HR	HR CI 95%	*p*-Value
Age (>60 year vs. ≤60 year)	2.5	1.1–5.7	**0.028**	3.3	1.2–8.9	**0.019**	1.6	0.6–2.2	0.542	1.3	0.5–3.1	0.616
Dural involvement (yes vs. no)	1.8	0.8–3.6	0.103	1.5	0.7–3.3	0.333	1.5	0.8–2.7	0.184	1.3	0.5–3.1	0.590
Surgery (CER/CFR vs. EER/OPF)	1.7	0.8–3.4	0.117	1.4	0.6–2.9	0.396	1.6	0.9–2.7	0.108	3.3	1.4–7.9	**0.006**
Margins (R1/2 vs. R0)	1.4	0.6–2.9	0.354	1.7	0.8–3.9	0.174	1.7	0.9–3.2	**0.062**	1.1	0.4–2.8	0.802

Abbreviations: CER, cranio-endoscopic resection; CFR, craniofacial resection; CI, confidence interval; DFS, disease-free survival; DSS, disease-specific survival; EER, endoscopic endonasal resection; HR, hazard ratio; OPF, osteoplastic flap; OS, overall survival; RFS, recurrence-free survival. Bold values are statistically significant.

## Data Availability

Data are available upon reasonable request.
